# Anomalous transparency in photonic crystals and its application to point-by-point grating inscription in photonic crystal fibers

**DOI:** 10.1038/s41598-018-23867-5

**Published:** 2018-04-03

**Authors:** Tigran Baghdasaryan, Thomas Geernaert, Karima Chah, Christophe Caucheteur, Kay Schuster, Jens Kobelke, Hugo Thienpont, Francis Berghmans

**Affiliations:** 1 Vrije Universiteit Brussel (VUB), Department of Applied Physics and Photonics (TONA), Brussels Photonics (B-PHOT), Pleinlaan 2, B-1050 Brussels, Belgium; 2 University of Mons, Electromagnetism and Telecom Department, B-7000 Mons, Belgium; 3 Leibnitz Institute of Photonic Technology, Albert-Einstein-Straße 9, D-07745 Jena, Germany

## Abstract

It is common belief that photonic crystals behave similarly to isotropic and transparent media only when their feature sizes are much smaller than the wavelength of light. Here, we counter that belief and we report on photonic crystals that are transparent for anomalously high normalized frequencies up to 0.9, where the crystal’s feature sizes are comparable with the free space wavelength. Using traditional photonic band theory, we demonstrate that the isofrequency curves can be circular in the region above the first stop band for triangular lattice photonic crystals. In addition, by simulating how efficiently a tightly focused Gaussian beam propagates through the photonic crystal slab, we judge on the photonic crystal’s transparency rather than on isotropy only. Using this approach, we identified a wide range of photonic crystal parameters that provide anomalous transparency. Our findings indicate the possibility to scale up the features of photonic crystals and to extend their operational wavelength range for applications including optical cloaking and graded index guiding. We applied our result in the domain of femtosecond laser micromachining, by demonstrating what we believe to be the first point-by-point grating inscribed in a multi-ring photonic crystal fiber.

## Introduction

Photonic crystals (PhCs) are man-made wavelength scale optical media enabling unprecedented control over the propagation of light^1^. It is well known that the periodicity of the dielectric function in such PhCs leads to the existence of so-called stop-band like ‘photonic band gaps’^2,3^, in which the propagation of light is prohibited in all directions within a certain frequency band. PhCs feature very anisotropic dispersive properties in the vicinity of the stop bands, resulting in remarkable phenomena such as negative refraction^4–6^, superprism effects^7^, self-collimation^8–10^ and others^1,11^.

However, for particular applications such as transformation optics^12–15^ and metamaterials^15–19^, one prefers working with PhCs that behave similarly to a homogeneous medium in terms of transparency and isotropy. Those typically work in the so-called ‘low frequency’ region, where the period and feature sizes of the PhC are much smaller than the wavelength of light, so that light propagating through the PhC experiences a spatially varying effective refractive index, as it essentially sees the spatial average of the structured medium instead of the individual domains of the lattice^16,17,20^. PhCs indeed usually behave as an isotropic homogeneous medium when they operate in the first frequency band below the lowest directional stop band. As soon as the wavelength becomes comparable with the period of the PhC, however, transparent behavior is no longer expected due to the appearance of strong resonant interactions between the electromagnetic wave and the periodic lattice of the PhC, and due to the strongly anisotropic nature of the lattice geometry. Note that when we use the term ‘transparent’, we refer to a behavior of the PhC that is similar to a homogeneous and isotropic dielectric, which allows for light to propagate without being scattered or diffracted, as for example through a simple glass slab.

In this paper and in contrast to what is commonly assumed, we show that for one polarization, PhCs can still be transparent for normalized frequencies as high as 0.9, i.e. well above the first stop band. This implies that the influence of diffraction is also minimal for these PhCs. We refer to this behavior as ‘anomalous transparency in PhCs’, since it appears at ‘anomalously’ high normalized frequencies in the proximity of the PhC’s resonance. We also show that for one polarization, such anomalous transparency in PhCs can be observed for a wide range of PhC feature sizes covering all filling factor values and within a relatively large normalized frequency bandwidth.

As a direct application of the finding, we tackled a long standing challenge relating to grating inscription in photonic crystal fibers (PCFs)^21^, and more specifically to micromachining^22^ or point-by-point grating writing^23–25^ in the core region of a PCF. Although such grating fabrication techniques have been demonstrated in standard optical fibers more than a decade ago^24^, they could not yet be applied to multi-ring PCFs due to the difficulties experienced with tightly focusing infrared femtosecond laser pulses through the microstructured cladding into the core region^26–31^. Using our findings we were able to design and to manufacture a PCF with a cladding that is transparent for the grating writing beam, and we inscribed what we believe to be the first infrared femtosecond laser pulse point-by-point Bragg grating in a multi-ring PCF^32^.

## Results

### Photonic band theory analysis

To introduce our approach, we first demonstrate that a PhC with specific feature sizes can behave in an isotropic manner for a range of normalized optical frequencies taken above the first directional stop band, using regular photonic band theory.

We do so by considering a two dimensional PhC that consists of a triangular lattice of air holes with a normalized radius of r/a = 0.42 in a background with refractive index of n = 1.45, and carrying out an eigenmode analysis of the PhC harmonic modes using the MIT Photonic-Bands (MPB) package^33^. Note that in order to study the dispersive properties of the PhC, we have to model the band diagram for the entire irreducible Brillouin zone (Γ-M-K-Γ).

**Figure 1:**
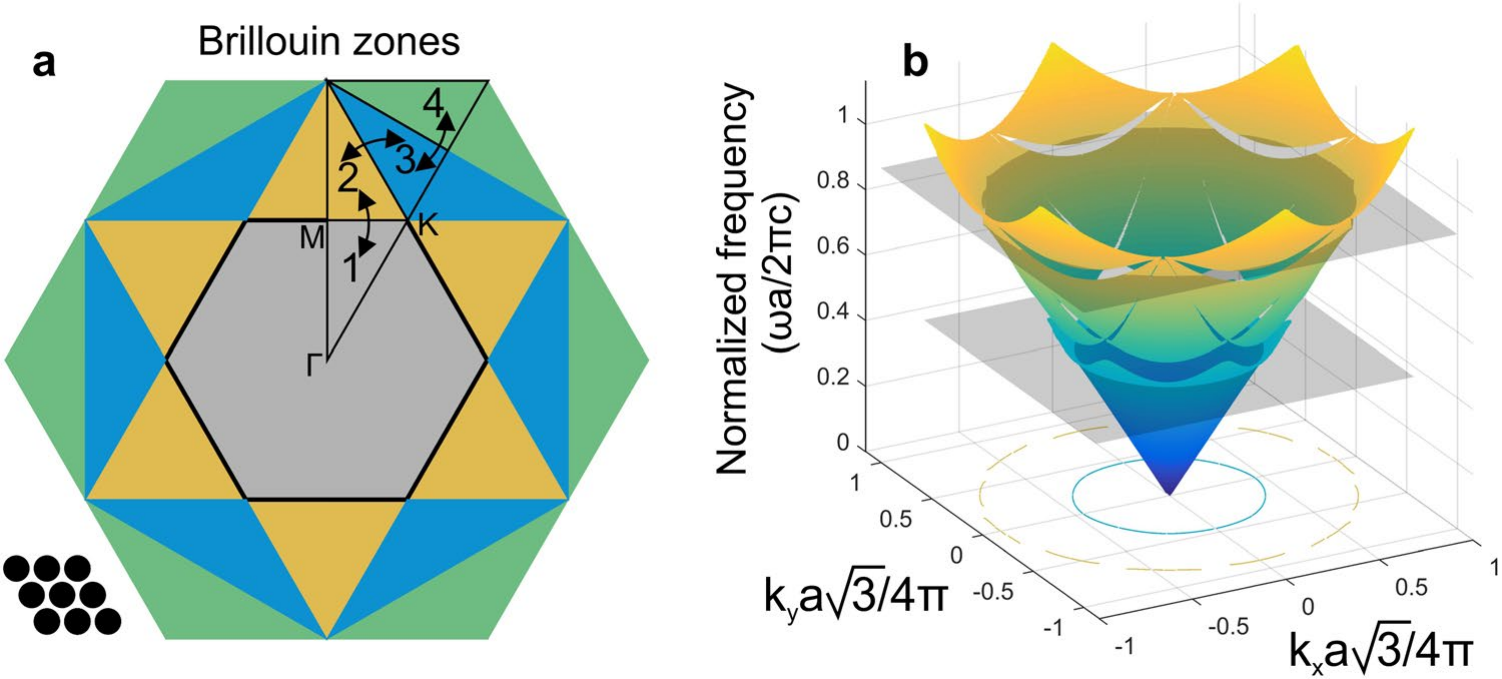
Unfolding procedure of Brillouin zones for a triangular lattice of air holes and photonic band diagram. (**a**) Illustration of the first four Brillouin zones and their unfolding procedure from the first irreducible zone for a triangular lattice shown in the inset. (**b**) Photonic band diagram of the two dimensional triangular lattice of air holes with r/a = 0.42 in a background with refractive index n = 1.45 for TE polarization.

The outcome of our simulations are the Bloch modes for several band orders, which are all folded into the first Brillouin zone and therefore need to be unfolded properly^34,35^. Fig. 1a shows the reciprocal space of a 2D triangular lattice and the unfolding procedure for the first four bands (shown with arrows). Different bands are indicated with corresponding numbers and colors. After unfolding the irreducible Brillouin zone, one can use the symmetry properties of the lattice to reconstruct the mode map for the complete Brillouin zones^36,37^ (see Fig. 1b). Although this essential procedure is well-known in the field of solid state physics^34^, it is often omitted in the photonics domain, which essentially complicates the interpretation of the dispersion properties using folded diagrams (as shown by Lombardet *et al*.^35^ for a rectangular lattice PhC). Proper unfolding leads to the photonic band diagram for the triangular air hole lattice depicted in Fig. 1b (for TE polarized light). This band diagram shows PhC modes for all propagation directions in 2D space depending on the normalized frequency (ωa/2πc or a/λ), where ω – is the angular frequency of light, λ – is the wavelength of light in vacuum, a – is the air hole pitch of the PhC lattice and c – is the speed of light in vacuum. The X and Y axes of the diagram correspond to the normalized components of the wave vector in the PhC. Fig. 1b, shows that different bands are separated from each other, forming directional stop bands. It is usually in the vicinity of those stop bands that the dispersive characteristics of the lattice are deformed.

**Figure 2:**
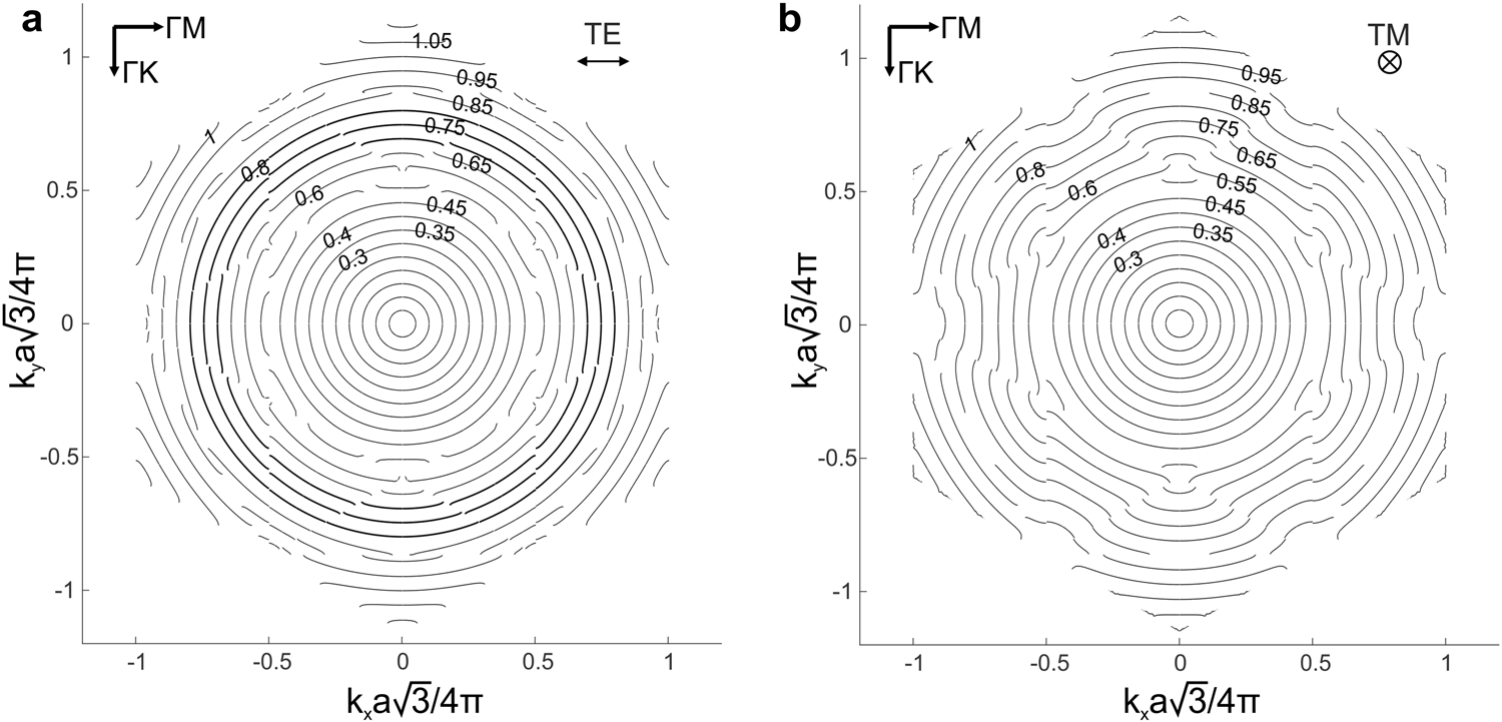
Isofrequency curves for the triangular lattice of air holes. Dispersive characteristics of the photonic crystal with r/a = 0.42 in a background with refractive index n = 1.45 for TE (**a**) and TM (**b**) polarizations. In the first figure the isotropic isofrequency curves at high frequencies above the first directional stop band are emphasized with a thicker line.

The angular dependence of the dispersive characteristics of the PhC that we are interested in becomes more obvious when we consider slices of the band diagram at a fixed frequency, to generate so-called ‘isofrequency’ curves. Fig. 1b illustrates such slices for normalized frequencies of 0.45 and 0.9 by means of two grey horizontal planes. More detailed isofrequency diagrams for both TE and TM polarized light are shown in Fig. 2a,b, where the frequency values are indicated next to the curves. Each curve reveals the entire set of possible wave vectors corresponding to particular Bloch modes of the PhC for a given frequency. For frequencies below 0.45, the diagrams are circular, meaning that the wave vector has the same length for all propagation directions and hence that the lattice behaves as an isotropic medium, as expected.

The first directional stop band is located around a normalized frequency of 0.5, which can be identified in Fig. 2 by pronounced irregularities in the isofrequency curves. For TE polarized light, the circularity of the isofrequency curves is remarkably restored for normalized frequencies from 0.7 to 0.8. The isotropy of the triangular lattice at such high frequencies for TE light is a first indication of anomalous transparency in this particular yet common type of PhC. The shape of the isofrequency curve is very close to an ideal circle at a normalized frequency 0.8, but a slight deviation from isotropy can be spotted when moving to lower frequencies in Fig. 2a. Indeed the isofrequency curve at a normalized frequency of 0.7, for instance, is slightly bent and has a small gap at the edge of the Brillouin zone. Depending on the incident light beam’s direction and on its angular spectrum, this deviation from a continuous and perfectly circular isofrequency curve will result in an undesired deflection of certain spatial frequency components. The presence of the gap implies that no Bloch modes are supported by the PhC in that particular range of wave vectors. However, because of the narrowness of the gap and hence of the very limited range of wave vectors for which this is the case, the overall impact on the transparency of the PhC in the mentioned isotropy region is limited. More detailed information on the refraction at the dielectric/PhC boundary can be found in the supplementary information to this paper (S1).

TM polarized light, on the other hand, shows clearly irregular curves above the stop band, in contrast to TE polarized light. Such different behavior of TE and TM polarizations in 2D PhCs is well known in photonic band theory^1^. It stems from the vector nature of the electromagnetic field and from the discontinuous boundary conditions at the interfaces between material and air. In our case of a 2D PhC, the electric field of TM polarized light is parallel to the interface of the air holes and remains unchanged when crossing this interface. For TE polarized light, however, the electric field is perpendicular to the interface and experiences a discontinuity when crossing the dielectric boundary.

Remarkably, the TE polarized light mode field distributions for different propagation directions in the triangular lattice in the second photonic band, also referred to as air band, have an almost identical effective index, which results in isotropy. A possible explanation for this can be the stronger localization in the low index region of the TE modes of the PhC in the air band, compared to the TM modes, which is again a consequence of the different boundary conditions^1^. As the low index region in our case is a circular air hole (which has an isotropic geometry by its nature), TE modes are more likely to feature the same effective index for different propagation directions.

### Anomalous transparency in photonic crystals

In the example above, we identified a PhC configuration with a normalized radius of r/a = 0.42 displaying isotropic behavior at anomalously high frequencies. In order for isotropy to be accompanied with transparency, we should make sure that the PhC does not scatter nor diffract light. To verify this, we use a Finite Difference Time Domain (FDTD) based approach to simulate the propagation of a tightly focused Gaussian beam through a PhC slab. If the PhC is also transparent, then it should allow focusing of an incident beam with high efficiency.

Such an approach can also be considered as an indirect way to identify transparent PhCs. The rationale is that if the PhC is isotropic and transparent, then it should not hinder tight focusing of the beam. Hence we also followed this approach to identify a set of PhCs that can behave in an isotropic manner and that are also transparent at such anomalously high normalized frequencies.

**Figure 3:**
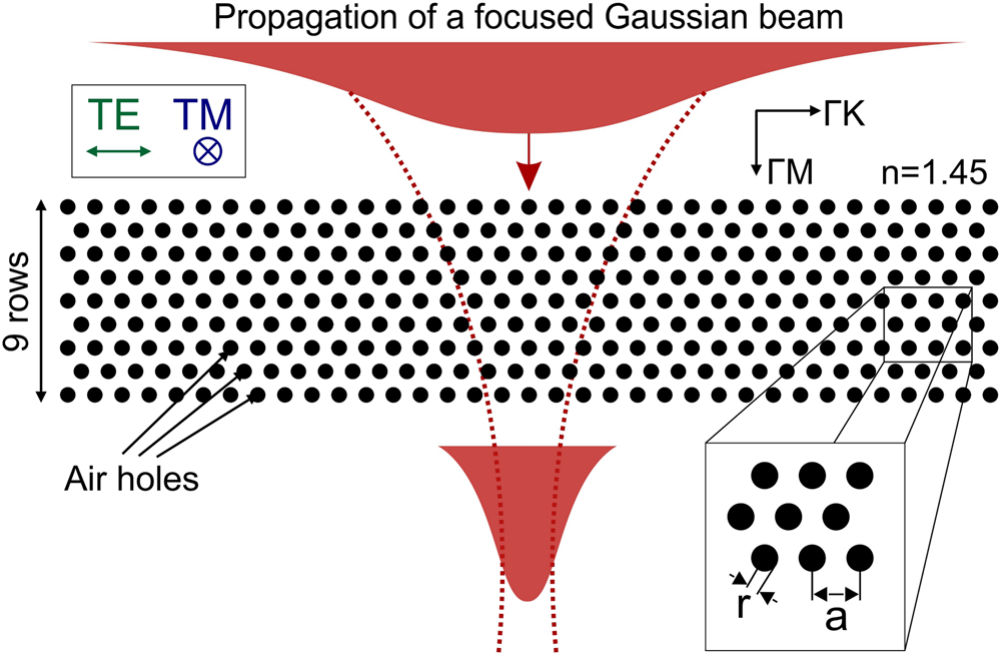
Modeling propagation of the focused Gaussian beam through a triangular lattice photonic crystal to identify transparent configurations. We consider a triangular lattice with 9 rows of air holes in a background with refractive index n = 1.45. Transparent photonic crystals with isotropic characteristics will allow efficient focusing through the photonic crystal. To model the dependence of the focusing efficiency on the lattice parameters, we start from a Gaussian beam that is focused with a lens with NA = 0.65 through the photonic crystal slab. The insets show the directions of the electric field oscillation for TE and TM polarized light, the ΓM and ΓK directions of the triangular lattice and the definition of the air hole radius ‘r’ and lattice pitch ‘a’.

The outline of the modeling methodology is shown in Fig. 3. The beam is incident along the ΓM direction of a triangular lattice PhC with 9 rows of air holes, in a background with a refractive index n = 1.45. We use a focusing lens with a numerical aperture (NA) of 0.65, which yields an angular spectrum for the incident Gaussian beam in the range from −26° to 26° in a homogeneous dielectric. As already stated, if the PhC behaves in an isotropic and transparent manner, it should not impede focusing through the structure, and the simulation should mimic focusing through a slab of a homogeneous dielectric material. The validation of this approach using isofrequency curves and the refraction phenomenon on the boundary of the PhC – homogeneous medium are presented in details as supplementary information to this paper (S1). In this supplementary information, we demonstrate that the angular spectrum of the focused beam inside the PhC covers two complete irreducible Brillouin zones inside the PhC, meaning that such a beam can excite all possible Bloch modes inside the PhC, taking into account the symmetry of the structure. Hence any deviation of the isofrequency curve from a circle will result in a decreased focusing efficiency. We recall that such an approach also allows estimating the influence of diffraction as well as judging on transparency rather than on isotropy only.

To quantify the transparency of the PhC slab, we consider the focusing efficiency as figure of merit, which is defined as the ratio of the maximal intensities in the focal region calculated with and without the holey structure. Efficiency values close to zero indicate that focusing is not possible, while values close to 1 refer to configurations that do not hinder tight focusing and that can hence be considered transparent.

**Figure 4:**
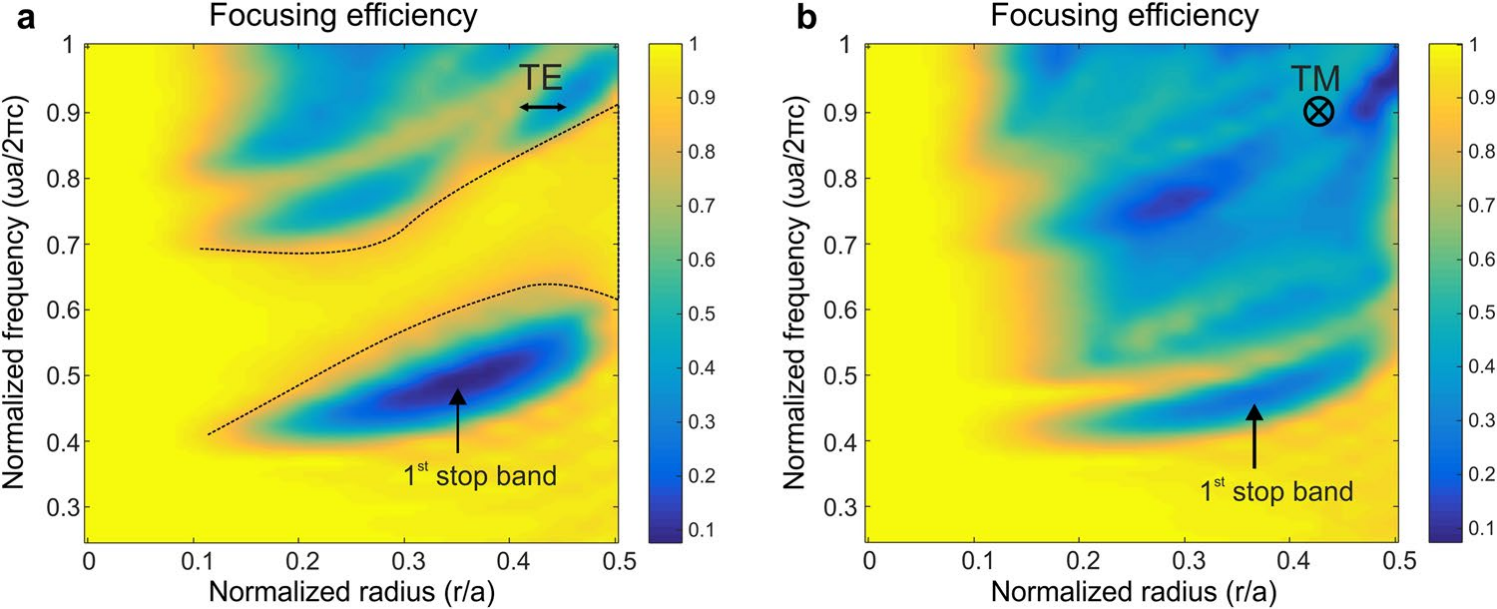
Parameterized study of tight focusing efficiency through a triangular lattice of air holes in a background with refractive index n = 1.45. The focusing efficiency is calculated as a function of the normalized radius and frequency for TE (**a**) and TM (**b**) polarized light, focused through a PhC with 9 rows of air holes along the ΓM direction of the lattice (shown in Fig. 3). A region of interest for TE polarization (**a**) with a high focusing efficiency corresponding to anomalous transparency region of the PhC is marked in between 2 dotted lines. For TM polarized light, no such region is observed.

Figure 4a,b show the focusing efficiency for TE and TM polarized light as a function of the normalized frequency (ωa/2πc or a/λ) and normalized radius (r/a), where r – is the radius of air hole. For both polarizations, efficient focusing is possible in the low frequency range (ωa/2πc < 0.4) or for small radii (r/a < 0.1). As stated before, these cases correspond to typical PhC lattice parameters for which light experiences the spatially averaged medium.

However, a remarkable region featuring focusing efficiencies around 1 appears for TE polarized light, for a relatively wide frequency range and for all possible filling factors. This region is clearly visible between the two dotted lines shown in Fig. 4a for anomalously high normalized frequencies. The focusing efficiency here is above 0.9, meaning that the transparency of the PhC for the focused beam is at least 90%. Such a wide parameter region above the first band, for which the PhC behaves like a transparent medium, has never been reported nor explored before, and we refer to it as a region of anomalous transparency. The region covers PhCs with any relative radius (filling factor) which implies a high degree of flexibility to realize PhCs for various applications, whilst leaving margin for fabrication errors and allowing for the conception of graded index structures. In addition, efficient focusing is possible for a wide range of normalized frequencies, which indicates that the use of broadband optical sources can be considered as well.

This region of anomalous transparency is located above the first directional stop band that can be observed as a blue region with very low focusing efficiency values just below the first dotted line. Note that the normalized frequency regions with high focusing efficiency for r/a = 0.42 above and below the first directional stop band are in agreement with the isofrequency curves that are circular as obtained in the previous section. Focusing efficiencies above 0.9 also mean that isotropy comes with transparency, and that diffraction is minimal in these regions.

The results for TM polarized light drastically differ from those for TE polarized light (see Fig. 4b). The known regions at low frequencies are present, but above the first directional stop band region the efficiency remains relatively low. The results are again in agreement with the behavior of the isofrequency curves for TM polarized light shown in Fig. 2b. We performed a similar study for normalized frequency values up to 3 for both polarizations, however we could not identify any other transparency region.

**Figure 5:**
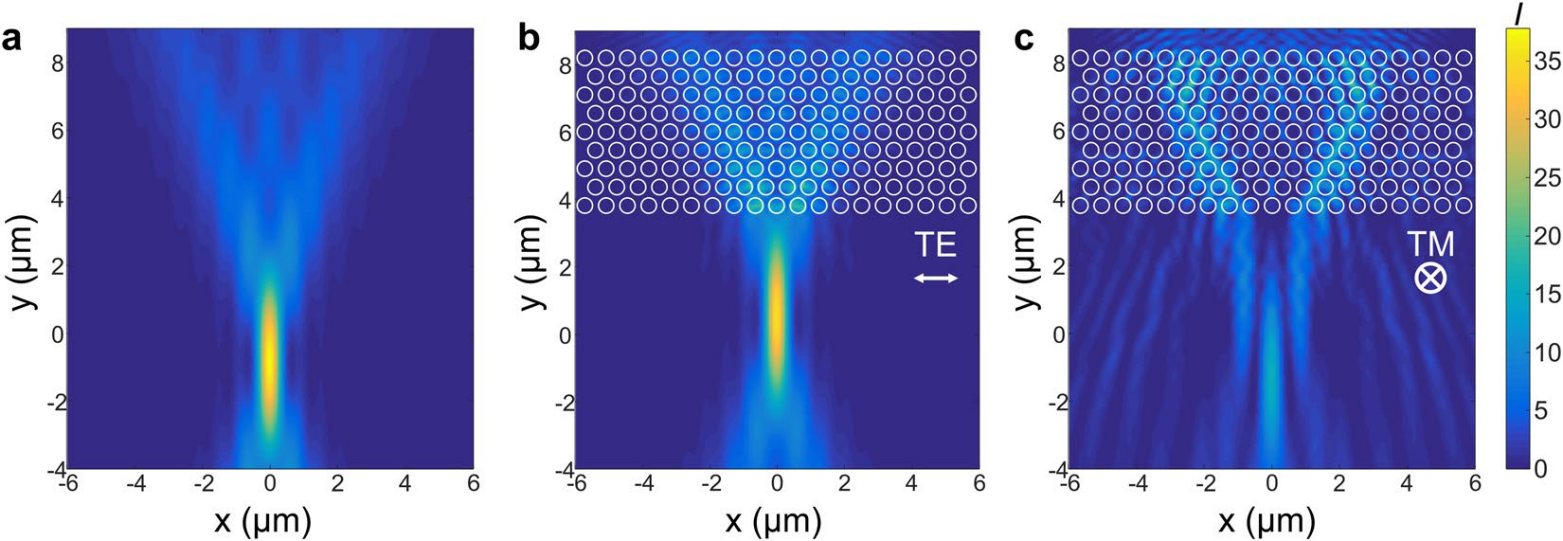
Tight focusing of a beam with NA = 0.65 lens in case of absence and presence of the photonic crystal for a normalized frequency of 0.8 to demonstrate anomalous transparency. Intensity distribution in the focal region in a dielectric with n = 1.45 when (**a**) no air holes are present. (**b**) When 9 rows of air holes are placed on the way of the focused beam for TE polarized light. Efficient focusing takes place owing to anomalous transparency of the photonic crystal at that normalized frequency. (**c**) The same as b. but now for the TM polarization. The focused beam is scattered due to the non-isotropic behavior of the photonic crystal.

To illustrate anomalous PhC transparency at relatively high normalized frequencies, we can look into results of the FDTD simulations for beam focusing through a PhC slab. We modeled propagation of the focused Gaussian beam in case of absence and presence of a PhC for TE and TM polarized light. Fig. 5a shows focusing of a beam with a lens with NA = 0.65 in a homogeneous dielectric with refractive index n = 1.45. Fig. 5b,c show the effect of a PhC with 9 rows of air holes and a normalized radius of r/a = 0.42, for a TE and TM polarized focused beam, respectively. Note that the wavelength and period of the grating are chosen to assure a normalized frequency of 0.8 (we used 800 nm wavelength in the FDTD simulation). For TE polarized light, the PhC lattice has a minimal influence on transverse focusing and the focal intensity distribution in this case is almost identical to that in the homogeneous medium (Fig. 5b). This simulation clearly demonstrates the transparency of the PhC for such a high normalized frequency. For TM polarized light, the converging phase front degrades due to the non-isotropic behavior of the PhC lattice, resulting in a highly scattered intensity distribution (Fig. 5c).

### Point-by-point grating inscription in photonic crystal fiber

The implications of the existence of anomalous transparency in PhCs can affect many application domains. Our finding indicates that the features of PhCs can be scaled up in a substantial manner and that their wavelength range of operation can be extended. This is also supported by the fact that we found anomalous transparency in PhCs for wide parameter and spectral ranges. One can consider demonstrations in the fields of transformation optics, metamaterials and graded index guiding when using PhCs as an effective index medium. However, in this section we will consider an application that provides a solution to a long standing challenge.

**Figure 6:**
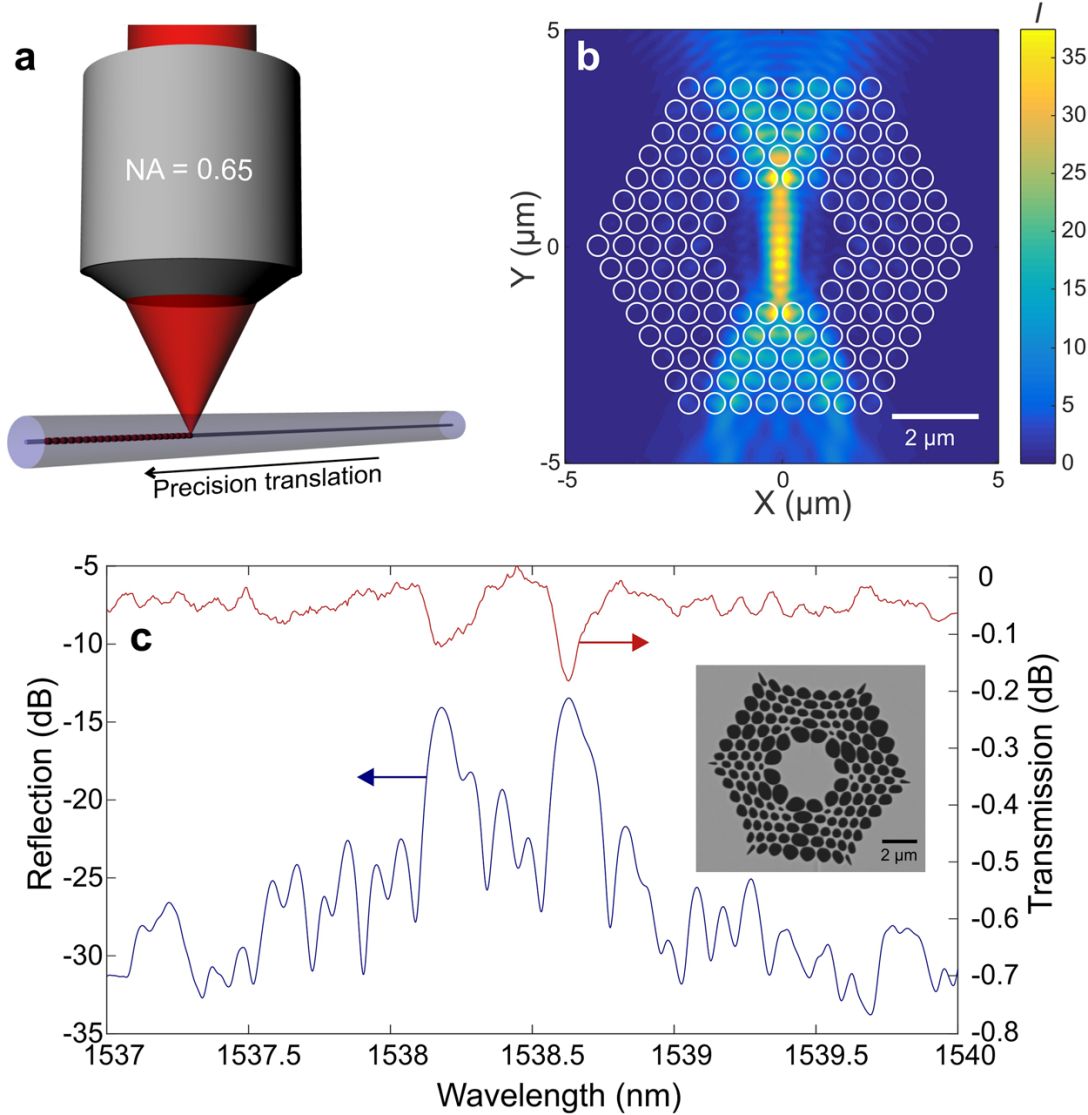
Point-by-point grating inscription in triangular lattice PCF with 5 rings of air holes in the cladding. (**a**) Outline of the point-by-point grating inscription approach using a high NA lens and fiber placed on a translation stage. (**b**) Design of the PCF and FDTD modeling result of the propagation of the TE polarized focused Gaussian beam, which demonstrates that high intensity regions are present in the core region and hence that the holey cladding has little influence on the focusing. (**c**) Reflection and transmission spectrum of the first point-by-point grating inscribed in the triangular lattice PCF with two distinct peaks (and valleys) resulting from the fiber birefringence. Scanning electron micrograph of the f.abricated PCF in the inset.

More specifically, we look into PCFs and femtosecond laser pulse based micromachining of fiber Bragg gratings (FBGs). Femtosecond laser pulse based grating inscription is schematically depicted in Fig. 6a. A laser beam is tightly focused into the fiber core region using a high NA objective lens^25^. In this configuration, the femtosecond laser pulses can modify the refractive index of the fiber core on the submicron scale as a result of non-linear absorption processes^22,23^. Compared with other methods for FBG fabrication, this direct writing approach eliminates the necessity of using a phase mask or an interferometer^23^ and allows rapid writing of complex grating structures such as chirped gratings^38^, phase shifted gratings^38^, plane-by-plane gratings^39^, birefringent gratings^25^ and superstructured gratings^38^ with controlled length and modulation^23^. Other advantages of the approach include the possibility to induce refractive index changes even in non-photosensitive fibers, as well as the ability to obtain gratings that remain stable up to temperatures of 1000 °C^40^, for applications in e.g. optical fiber lasers^41^ or optical fiber sensors^42^.

However, when the fiber is a PCF and therefore structured by means of a PhC in its cladding, micromachining becomes a challenge as the photonic structure impedes efficient focusing of the laser beam^26^. The only report of point-by-point FBG inscribed in a glass microstructured fiber was in a highly birefringent Germanium doped PCF with only three rows of air holes^31^. Our objective here is to exploit the existence of anomalous transparency in a PhC when applied to a PCF, in order to enable direct FBG writing using an 800 nm femtosecond pulse laser in a traditional hexagonal shape multi-ring PCF. Working in the infrared region rather than with ultraviolet light conveys the advantage that the PhC can feature even larger dimensions, which favors its manufacturability.

To do that we designed and fabricated a dedicated PCF. The lattice parameters of the PCF cladding are a pitch a = 0.6 µm and an air hole radius r = 0.25 µm. This corresponds to a normalized radius r/a = 0.42 and a normalized frequency of 0.75, which lies in the middle of the identified PhC transparency region presented in Fig. 4a.

The cross section of this dedicated PCF is illustrated in Fig. 6b. The core region is formed by omitting 3 rings of air holes in the center of the triangular lattice, and the cladding region consists of 5 rings of air holes. The core diameter of the PCF is around 3 µm, while the outer cladding diameter is 125 µm. The PCF is designed to be single mode around 1550 nm.

Figure 6b also shows the modeled intensity distribution in the PCF cross-section when a Gaussian beam at 800 nm is focused with a NA = 0.65 lens to the core region. As anticipated, the triangular lattice cladding appears not to prohibit transverse focusing of the beam owing to the isotropic behavior of the PhC with these particular features. Moreover, for this PCF, control over the orientation of the triangular lattice cladding relative to the writing beam direction is no longer an issue owing to the isotropy of the lattice.

The fabrication of such a fiber in pure silica material was made possible with a stack-and-draw technique^43^, in spite of the sub-micrometer sizes of the air holes^44^. A scanning electron micrograph of the cross-section of the fabricated fiber is shown in the inset to Fig. 6c. While the filling factor is large, as targeted, the sub-micrometer air hole dimensions vary considerably across the lattice. The air hole pitch lies between 0.6 µm − 0.9 µm and is slightly larger than designed, while the cladding diameter is 125 μm. Our simulations show that due to the asymmetry of the holey cladding, the PCF displays a phase modal birefringence of 5 × 10^−4^ at 1550 nm.

Despite the deviation of the fabricated PCF from the initial design parameters we successfully micromachined a 4th order FBG with a period of 2.18 µm using a 800 nm fs point-by-point grating inscription setup^25^.

Figure 6c shows the reflection spectrum of the grating measured with unpolarized light with absolute reflection values recalculated from the measurements of the transmission spectra. Two clear grating peaks are visible with a peak separation of 0.45 nm, which is consistent with the simulated phase modal birefringence of the PCF. Each peak reflects around 4.5% (corresponding to −0.2 dB transmission calculated for the second dip) of the light confined in the fundamental mode. To the best of our knowledge this is the first point-by-point fiber Bragg grating inscribed in a pure silica multi-ring PCF through a structured cladding, which provides experimental support to the existence of anomalous PhC transparency, meaning in the high frequency region of a PhC.

## Discussion

To conclude, we have demonstrated that a PhC can behave as a transparent and isotropic medium at much higher normalized frequencies than reported before. We developed a methodology to identify transparent PhC configurations based on photonic band theory and supplementary FDTD simulations of the propagation of a tightly focused beam through a PhC slab. We applied this approach to a triangular lattice of air holes in a background with refractive index n = 1.45. For TE polarized light, we identified a wide range of transparent PhCs above the first directional stop band for normalized frequencies as high as 0.9. The influence of diffraction was minimal in this range as we achieved high focusing efficiency through a PhC slab with 9 rings of air holes.

Our finding allows upscaling PhC features and hence easing their manufacturing or lowering their operational wavelength. 3D printed metamaterials^17,19,45,46^, graded index photonic crystals^13,14,47^ and optical cloaks^16,17,48^ at visible wavelengths are within reach owing to such isotropic PhCs with much larger features. Given that the refractive index of the material that we used in our simulation is close to that of polymers, our findings can impact the application range of polymer nano-imprinting technologies as well^17,45,46^. Finally, we identified a new family of PCFs with a cladding that is transparent for transversely propagating light and that can be designed to serve applications ranging from fiber lasers^42^ to fiber sensing and lab-in-fiber^49^. We demonstrated this with the first point-by-point femtosecond laser written FBG in a dedicated triangular lattice multi-ring PCF^32^.

## Methods

### Numerical modeling

We used a finite difference time domain (FDTD) method implemented in Lumerical FDTD Solutions software^50^ to model the propagation of the focused beam through the photonic crystal lattice in pure silica (refractive index n = 1.45). We considered an incident wavelength of 800 nm. To study the influence of the triangular lattice photonic crystal on focusing efficiency, we modeled the incidence of a Gaussian beam focused with a NA = 0.65 lens, and we surrounded the simulation region with a perfectly matching layer. For our eventual analysis, we recorded the intensity distribution in the focal region with a frequency domain monitor. To model the intensity distribution in the PCF cross-section, we used identical modeling conditions. Note that the influence of the outer cladding of the fiber was not taken into account.

The waveguiding properties of the PCF were modeled using Lumerical Mode Solutions software at a 1550 nm wavelength. The designed fiber is single mode at this wavelength and has an effective index of 1.4. We calculated that the confinement loss of the PCF is of the order of 0.1 dB/m. For the as-built PCF, we used the geometry extracted from the SEM image of the cross-section. The confinement loss for the fundamental mode was simulated to be around 2 × 10^−3^ dB/m. The effective index of the fundamental mode was 1.4. Due to the asymmetry of the air holed cladding, the PCF is (highly) birefringent with a phase modal birefringence of 5 × 10^−4^.

### Photonic crystal fiber (PCF)

The triangular lattice PCF has been prepared using a multi-step stack-and-draw approach^43^. All used rod and tube materials were made from Heraeus Suprasil F300 silica glass. In the first step, 163 glass rods with a diameter of 1000 µm were drawn from a pure silica preform. In the second step a silica tube (outer and inner diameter 25 and 19 mm) has been drawn to 150 thin-walled capillaries. The inner pressure has been controlled to keep the capillary diameter constant at values of 1000 µm (outer diameter) and 778 µm (inner diameter), respectively. The drawing has been followed by a manual stacking of the individual elements in a triangular arrangement of a 10 ring structure. This stacked package was overcladded with a jacket tube (outer and inner diameter 30 and 19.5 mm) and drawn under a temperature of 1940 °C and thus stretched to an elongated cane (outer diameter 5.9 mm). The main challenge was the uniform fusing of all glass components to a dense structure while maintaining the triangular lattice. To meet this demand, the package arrangement was fused at both ends, enabling the passive formation of an internal excess pressure. During the cane drawing, the excess pressure inflates the capillaries while the interstitial volume was evacuated by an appropriate vacuum. In the final manufacturing step, the cane was overcladded by an additional jacket tube (outer and inner diameter 30 and 6 mm) and drawn (T ≈ 2020 °C) under differential pressure conditions to obtain the eventual PCF (outer diameter 125 μm). To this end the cane geometry inflates by defined pressurizing, leading to the final microstructured fiber.

### Point-by-point grating inscription

To produce the grating in the PCF, we have used the direct point-by-point inscription technique with 800 nm femtosecond laser pulses (120 fs pulse duration and 1 kHz repetition rate). Details on the experimental setup and inscription process have been described in previous work^25^. The laser beam is delivered by a Spitfire pro Spectra-Physics amplifier, is attenuated down to 200 nJ (pulse energy) and is tightly focused into the PCF core region with a high numerical aperture (0.65) microscope objective without oil immersion. The PCF is maintained over a high-precision air-bearing translation stage and displaced along its optical axis with a controlled speed, so that each single focused femtosecond pulse induces a local (point-like) refractive index modification. To create a regular point-like spacing and thus a uniform grating with a specific period over the required length, we applied a constant displacement speed matched to the repetition rate of the laser.

## Electronic supplementary material

Supplementary Information
