# Supplementary material for: Anomalous transparency in photonic crystals and its application to point-by-point grating inscription in photonic crystal fibers
Source: Sci Rep. 2018 Apr 3;8:5470. doi: 10.1038/s41598-018-23867-5 (PMC5882901; doi:10.1038/s41598-018-23867-5)
Supplement: Supplementary file 1 — Supplementary Information [file 41598_2018_23867_MOESM1_ESM.docx]

Anomalous transparency in photonic crystals and its application to point-by-point grating inscription in photonic crystal fibers

Tigran Baghdasaryan^1,*^, Thomas Geernaert^1^, Karima Chah^2^, Christophe Caucheteur^2^, Kay Schuster^3^, Jens Kobelke^3^, Hugo Thienpont^1^ and Francis Berghmans^1^

^1^Vrije Universiteit Brussel (VUB), Department of Applied Physics and Photonics (TONA), Brussels Photonics (B-PHOT), Pleinlaan 2, B-1050 Brussels, Belgium

^2^University of Mons, Electromagnetism and Telecom Department, B-7000 Mons, Belgium

^3^Leibnitz Institute of Photonic Technology, Albert-Einstein-Straße 9, D-07745 Jena, Germany

*tbaghdas@vub.be

**Supplementary information**

**Refraction of a tightly focused beam at the interface between a homogeneous dielectric and a photonic crystal**

To validate the use of the focused Gaussian beam using a lens with NA=0.65 for identifying isotropic and transparent PhCs with our FDTD simulations, we demonstrate that the angular spectrum of the focused beam covers two complete irreducible Brillouin zones. This means that, for incidence along the ΓM axis, such a beam excites all possible Bloch modes inside the PhC taking into account the symmetry of the structure.

We limit the discussion to the case of a normalized frequency of 0.8, for which the isofrequency curve is closest to a perfect circle for TE polarization. As shown in Fig. S1a, for a given k-vector incident under a certain angle θ1, there are reflected and refracted rays similar to what happens at the interface between non-structured dielectric media. The direction and magnitude of the refracted k-vector in the PhC for a given incident k-vector can be found using the isofrequency curves for TE and TM polarized light, as illustrated in Fig. S1b and S1c, respectively. To identify the refracted wave vector inside the PhC, one should first draw the isofrequency curve of the incident medium (ideal semicircle), which is an isotropic homogeneous dielectric with n=1.45. The diameter of this semicircle is given by the refractive index of the medium and the normalization procedure of the k-vector used for the triangular lattice. The next step consists in drawing the actual isofrequency curve of the PhC for a normalized frequency of 0.8, as obtained from Fig. 2. For a given angle of incidence θ1, one can use the required conservation of the wave vector component parallel to the interface between the two media to find the corresponding wave vector in the PhC. The wave vector of the refracted beam in the PhC is then found on the isofrequency curve of the PhC for the same parallel wave vector component as the incident beam.

Owing to the specific shape of the isofrequency curves, a PhC can behave in the same way as a classical anisotropic medium, in which the direction of propagation of the energy differs from the wave’s direction of propagation, i.e. from the direction of the phase velocity. This has implications on tight focusing of a beam through a PhC slab as we describe below.

The direction of the energy propagation in PhCs corresponds to the direction of the group velocity, which is perpendicular to the isofrequency curves. Directions of the group velocity for different incident angles are shown in Fig. S1 with red arrows. The phase velocity determined by the wave vector and the energy propagation direction in the PhC coincide for all orientations only in the case of a circular isofrequency curve, i.e. in the case of an isotropic medium. As clarified in Fig. S1b, for TE polarized light and a normalized frequency of 0.8, the isofrequency curve of the PhC almost coincides with an isofrequency curve for an isotropic medium shown with a black dashed line. However, for TM polarized light shown in Fig. S1c, the picture is different as the magnitude of the wave vector depends on the orientation, and the direction of propagation of the electromagnetic energy is different from that of the phase velocity for the majority of orientations.

If we consider focusing of a beam through such a PhC, then the angular spectrum of the beam includes a wide range of incident wave vectors. For a lens with NA=0.65 and n=1.45, the beam holds k-vectors with angles from -26° to 26°. From the isofrequency curves depicted in Fig. S1, we find that the angular spectrum of the k-vector extends from -34° to 34°inside the PhC lattice, which covers the entire angular range of two irreducible Brillouin zones (that extend from -30° to 30°). Hence, given the symmetry of the triangular lattice and the fact that the beam is incident along the ΓM direction, such a focused beam excites all the Bloch modes inside the PhC lattice. All the excited Bloch modes will propagate inside the PhC according to the scheme depicted in Fig. S1 and described above. In case of non-circular isofrequency curves, different Bloch modes will experience a different phase delay due to a difference in the absolute value of their wave vector (and hence effective index) and a difference in their propagation direction, as well as in their phase velocity directions. Due to these effects, a converging phase front that enters the PhC slab will be distorted and one will not observe perfect focusing of the beam. This is, in particular, very likely to happen for TM polarized, while for TE polarized light we do not expect distortion of the focused phase front (apart from diffraction).


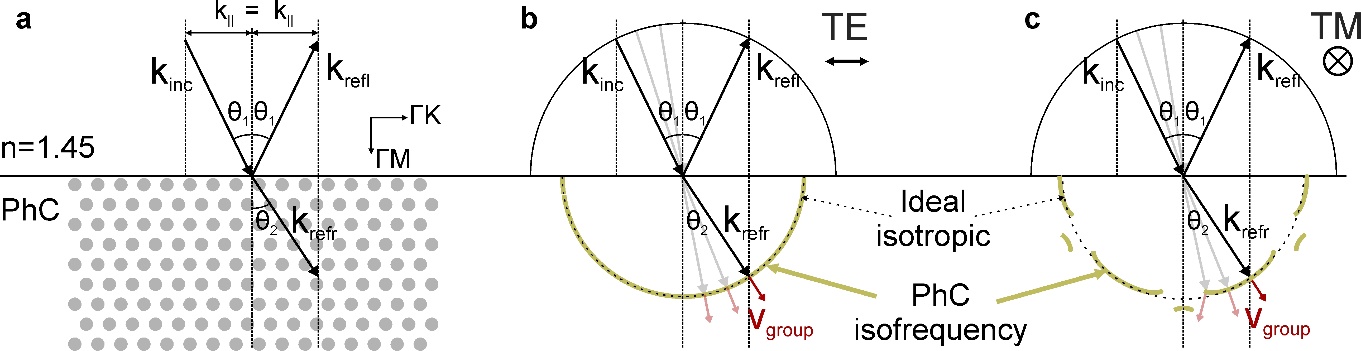


**Figure S1.** Refraction at the interface of a dielectric and photonic crystal for light with normalized frequency of 0.8. (a) General refraction and reflection scheme with emphasis on the conservation of the wave vector component parallel to the interface. We also outline how the wave vector and group velocity direction are identified inside a photonic crystal using the isofrequency curves for TE (b) and TM (c) polarizations.
